# Investigations into the Material Characteristics of Selected Plastics Manufactured Using SLA-Type Additive Methods

**DOI:** 10.3390/polym16111607

**Published:** 2024-06-06

**Authors:** Dominika Grygier, Adam Kurzawa, Mateusz Stachowicz, Karina Krawiec, Maksymilian Stępczak, Maciej Roszak, Mikołaj Kazimierczak, Dorota Aniszewska, Dariusz Pyka

**Affiliations:** 1 Department of Vehicle Engineering, Faculty of Mechanical Engineering, Wroclaw University of Science and Technology, Smoluchowskiego 25 Str., 50-370 Wroclaw, Poland; 2 Department of Lightweight Elements Engineering, Foundry and Automation, Faculty of Mechanical Engineering, Wroclaw University of Science and Technology, Smoluchowskiego 25 Str., 50-370 Wroclaw, Poland; adam.kurzawa@pwr.edu.pl (A.K.); mateusz.stachowicz@pwr.edu.pl (M.S.); 3 Department of Mechanics, Materials and Biomedical Engineering, Faculty of Mechanical Engineering, Wroclaw University of Science and Technology, Smoluchowskiego 25 Str., 50-370 Wroclaw, Poland; 254762@student.pwr.edu.pl (K.K.); 252330@student.pwr.edu.pl (M.S.); maciej.roszak@pwr.edu.pl (M.R.); 255121@student.pwr.edu.pl (M.K.); dorota.aniszewska@pwr.edu.pl (D.A.); dariusz.pyka@pwr.edu.pl (D.P.)

**Keywords:** additive manufacturing, stereolithography, resins, mechanical properties, constitutive models, SEM analysis, biological properties

## Abstract

In this study, the authors performed a strength analysis of seven groups of commercially available materials based on SLS incremental technology. Test samples were made with Original PRUSA SL1S printers, with 10 samples of each type from 7 resins selected for testing. The tests were carried out on an MTS Bionix machine in a static tensile test, during which the basic mechanical properties were determined. This is also a preliminary study to determine material constants in the Johnson-Cook strength model. The authors then performed numerical simulations to mirror the experimental tests in order to tune the rheological model. In addition, a fracture criterion was determined based on a hybrid FEM/SPH numerical method. This allowed for the expansion of material libraries currently used in numerical simulations, as well as the sensitivity of the materials’ models. In subsequent studies, in order to determine the nature of material destruction, analysis of fracture surfaces was performed using a scanning electron microscope (SEM). The final study was a biocompatibility test to assess the biological properties of the material. The conducted research made it possible to determine the strength properties of resins currently used in 3D printers, expand the libraries of material models in the computational environment (with an error rate of less than 5%), as well as observe the nature of the cracks formed and biocompatibility in the context of predicting the use of these materials for biomedical applications.

## 1. Introduction

Additive manufacturing, increasingly recognized as a transformative production technology, plays a pivotal role in various industries due to its efficiency, cost-effectiveness, and capacity for complexity without additional waste [1,2,3,4,5,6,7]. Among the primary methods of additive manufacturing, stereolithography (SLA) is distinguished by its superior surface quality and precision, making it especially suitable for applications in dentistry, where detail and accuracy are most important [8,9,10,11].

SLA operates on the principle of polymerizing liquid resin with a UV light, a technique that enables the production of parts with sophisticated details and smooth finishes. This method is contrasted with selective laser sintering (SLS) and fused deposition modeling (FDM), which involve the sintering of polyamide-based powders and the layering of thermoplastic materials, respectively. While SLS is known for its strength and impact resistance. FDM, although economical, typically produces parts with a lower resolution and surface quality compared with SLA. In a study by Mi-Young Sim et al. [12], it was shown that of the three additive manufacturing methods analyzed, SLA showed the highest accuracy in terms of intercoating deviation. This precision is particularly important, especially in the context of the potential application of SLA to produce implants for the human body. Ensuring a high degree of accuracy can influence the effectiveness, safety, and minimization of the risk of implant-related complications. Thus, knowing the material parameters of the resins used in the SLA method is crucial to achieving the desired results.

In 2015, Tumbleston et al. [13] made a significant advancement in stereolithography with the introduction of a novel third-generation technology. Their development, known as Continuous Liquid Interface Production (CLIP), has revolutionized the field, enabling the printing process to be significantly faster than previous methods. This breakthrough allowed components to be produced in just a few minutes, a significant improvement on previous standards, where several hours were required. These results show that SLA 3D printing technologies create models with satisfactory dimensional accuracy for surgical use in a rather short span of time, which gives reason to further explore the materials used in this method in order to further develop this technology.

The introduction of Multi Jet Fusion (MJF) has provided an alternative by sintering metal powders using a focused laser, which facilitates even greater structural integrity. However, SLA remains preferred for producing transparent and finely detailed parts due to its precision. The discussion in this paper will focus on SLA while referencing comparative data to highlight its benefits over other methods [14,15,16,17].

Recent advancements in 3D printing have been catalyzed by the development of innovative materials such as novel resins, which are crucial for expanding the capabilities of technologies like SLA [15,16,17,18,19,20,21]. Research such as that conducted by Szykiedans et al. [22] has shown variations in the mechanical properties of these materials, influencing the choice of printing parameters. Szykiedans et al. conducted strength tests on materials such as ABS, Z-Glass, and photopolymer resin for Nobel printers, revealing differences in the Young’s modulus between the 3D prints and their base materials. For example, the Z-ABS prints showed an average Young’s modulus of 1.12 GPa, while the encyclopedic value ranges from 1.7 to 2.1 GPa. The Young’s modulus was 1.43 GPa for Z-Glass and 246 MPa for the Nobel printer photopolymer resin, indicating significant discrepancies due to factors like manufacturing parameters or apparatus accuracy. This indicates a further need to catalog and document the mechanical properties of the materials in question in order to base them on the values that are most likely to be obtained in most cases.

Orientation and layer thickness have also been found to significantly affect the mechanical behavior of printed objects, emphasizing the need for detailed parameter optimization to achieve desired outcomes. The study by Saini et al. [23] included five different layer orientations—0°, 22.5°, 45°, 67.5°, and 90°—with findings that the orientation plays a crucial role in the mechanical properties of prints. The specimens printed at 22.5° and 67.5° demonstrated the highest tensile and compressive strength, information that was instrumental in the design of specimens for this research to ensure uniform and reproducible results.

Furthermore, as the field of additive manufacturing evolves, the application of finite element analysis (FEA) has become increasingly important in predicting the performance of printed parts [24,25,26,27]. Supported by constitutive models developed by researchers such as Wang et al. [28], who derived an elastoplastic constitutive model, and Sælen et al. [29], who developed a hyperelastic model with a brittle criterion, FEA provides valuable insight into the potential applications and limitations of SLA-printed materials. These models are implemented in commercial finite element analysis software and tailored for material testing, increasing our understanding of the behavior of materials under different conditions and aiding in the optimization of the printing process, as well as providing reliable data in the form of constitutive models that can be implemented for the calculation of various types of components and entire structures.

## 2. Materials and Methods

### 2.1. Characteristics of Printed Materials

The materials tested were selected plastics commonly used in 3D printing technology. Masked stereolithography (MSLA) additive manufacturing was used in the study. The set included seven different materials belonging to the resin group. The materials analyzed were characterized by varying parameters, including density and hardness.

**Figure 1:**
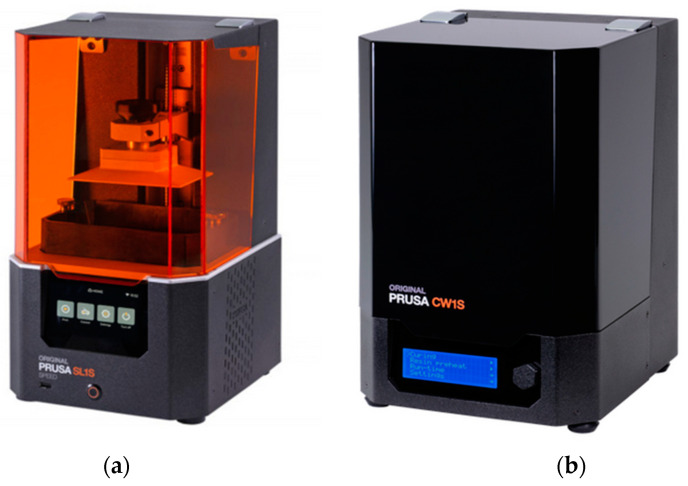
Equipment used: (**a**) PRUSA SL1S printer and (**b**) Original Prusa Curing and Washing Machine CW1S (Czech Republic, Prusa Research) [30].

All material samples were produced at a 90° angle to the printing plane. Table 1 shows the details of the selected resin types, together with the mechanical property data provided by the manufacturers. These material samples were intended to be printed with the Original Prusa SL1S 3D printer (Praha, Czech Republic, Prusa Research) (Figure 1) using MSLA technology.

The Original Prusa SL1S printer has been factory calibrated to work with resins with a curing wavelength of 405 nm. It is possible to use different resin brands, as long as they comply with the light wavelength requirements. Compared with the traditional FDM method, the products obtained with the described technology have a higher surface quality (no visible layers) and a higher level of detail.

The first material used was ABS-Like from Anycubic (Shenzhen, China, Anycubic), characterized by excellent tensile and flexural strength due to the presence of polyurethane acrylate. The material also features a high hardness and better flowability compared with conventional resins, resulting in shorter curing times during printing. Its curing wavelength is 365–405 nm, and its minimal shrinkage contributes to a print precision of ±0.1 mm, ensuring smooth surfaces and dimensional stability for printed objects.

The second material used was AnyCubic’s 3D Printing UV-Sensitive Resin, based on the standard colored UV resin formula. It is characterized by high strength, low shrinkage (3.72–4.24%), and good adhesion. It delivers impressive print results in terms of success and accuracy as well as stability in humid and corrosive environments.

The third material was the newly developed Anycubic UV Tough, combining hardness, elasticity, resistance to mechanical damage, and low shrinkage. It is characterized by high precision and strength, according to the set parameters for 3D printers.

ECO UV Resin from AnyCubic, a vegetable resin based on soybean oil, was used as the fourth material, characterized by the absence of harmful chemicals and high-quality and precision prints.

DLP Craftsman Resin from AnyCubic was the fifth material, characterized by low shrinkage and high precision as well as the ability to print quickly.

The 3D Rapid resin from Monocure (San Leandro, CA, USA, Monocure 3D) was the sixth material, showing extremely fast polymerization and minimal shrinkage with no VOC emissions.

The last material used was Prusament Resin Tough (Praha, Czech Republic, Prusa Research), a top-quality resin that is easy to print, precise, and safe for the user, although characterized by low temperature resistance and a lack of liquid recycling.

These materials were selected based on their popularity and availability and to complement the information on their physical properties.

### 2.2. Testing of Materials Using a Static Tensile Test

**Figure 2:**
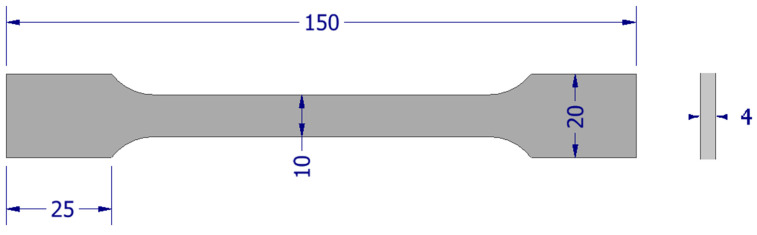
Shape adopted for the static tensile test.

According to ISO 527-3:2019-01 [31], concerning plastics and the determination of their mechanical properties in the static tensile test, certain conditions and procedures were adopted for carrying out this test. The standard establishes a standard shape for the specimen, which takes the form of a ‘paddle’. The main dimensions of this specimen were an overall length of 140 mm, length of the gauge section of 90 mm, width of the gauge section of 10 mm, and thickness of 4 mm, with an accuracy of ±0.2 mm. Figure 2 shows the adopted shape along with the dimensions, which were scaled to consider technological requirements.

The test procedure consisted of progressively stretching a carefully prepared flat specimen at a set constant speed. For this process, a testing machine was used which was equipped with suitable jaws for clamping the specimen, a dynamometer for measuring the force applied to the specimen, and a displacement sensor to record the elongation Δl relative to the initial length of the specimen.

By analyzing the recorded values of the force F and the elongation Δl of the specimen, the characteristics σ = f(ε) for the test specimens could be obtained. The shape of the resulting curve depended on the type of material, which made it possible to infer its mechanical properties.

**Figure 3:**
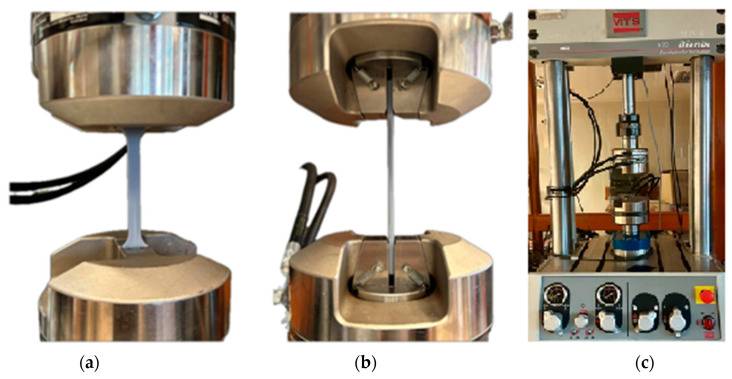
Specimen placement: (**a**) front view; (**b**) side view; and (**c**) Bionix MTS testing machine used for testing.

A Bionix MTS (Figure 3) testing machine was used to carry out the test. Jaws specific to tensile testing were used to clamp the specimens. A jaw travel speed of v = 5 mm/s was chosen for a maximum distance between the jaws of 50 mm, which represented the maximum elongation length. Five specimens of each material were prepared (see Table 1), and the specimens were placed symmetrically with respect to the grips. One tensile test was carried out for each specimen, and the test was stopped when the test specimen lost continuity.

### 2.3. Methodology of Numerical Simulation

**Figure 4:**
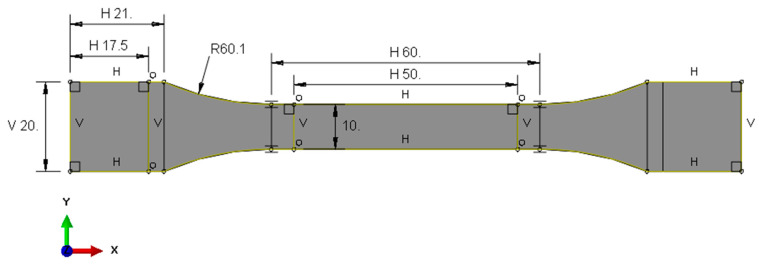
Geometry of paddle sample in numerical environment (Abaqus).

The aim of the work was to determine the tensile strength of 7 groups of resins used for 3D printing. At the same time, the authors’ goal was to develop numerical models that could, in the future, support the processes of design, construction, and analysis of the behavior of elements that would be printed using this material database. For this purpose, a number of numerical simulations were also carried out to develop numerical models of the materials. For this purpose, first, a numerical model of the paddle sample was developed in the Abaqus computing environment. The numerical model of the sample based on the actual dimensions of the sample is presented below (Figure 4).

**Figure 5:**
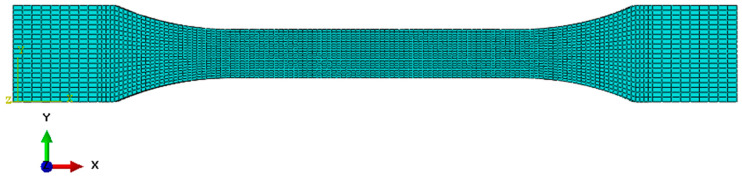
Finite element meshed sample in the Abaqus/Explicit computing environment.

Then, the sample was discretized with hex elements from the Explicit library in the Abaqus/Explicit computing environment. The size of the finite elements was 2.0 mm in the mounting places and 1 mm in the central part of the sample. The tested sample with a finite element mesh applied is shown below (Figure 5). The finite element mesh shown was selected based on the mesh sensitivity analysis performed. Finally, because of the mesh selection method, the element in the area of rupture occurrence had dimensions of 1 mm × 1 mm × 0.5 mm. The total number of finite elements was 10,880.

**Figure 6:**
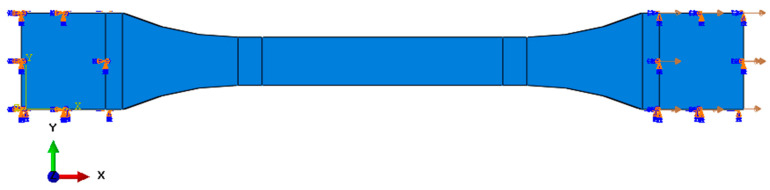
Sample with initial boundary conditions.

For the lower surface of the sample, the X, Y, and Z axis translational and rotational degrees of freedom were removed, which corresponded to the stationary mounting location. On the second surface, all rotational degrees of freedom and translational degrees of freedom in the Y and Z axes were deprived. The translation in the X axis remained unlocked, which allowed the testing machine to operate at a feed speed of 5 mm/s. The sample with the given initial boundary conditions is presented below (Figure 6).

The Johnson-Cook constitutive model was used to describe the material’s behavior in the plastic range. This model is quite popular due to the relatively simple determination of material parameters but also the presence of this formula in many numerical calculation programs [32,33,34]. Due to the lack of tests related to the influence of the strain rate and temperature on the strength of samples, only the first part of the formula—related to plastic strengthening—was used:

(1) $$\sigma_{y}=\left(A+B{\underline{\varepsilon }}^{p^{n}}\right)\left(1+Cln{\dot{\varepsilon }}^{*}\right)\left(1-T^{*m}\right)$$

where *A* is the yield strength, *B* is the strengthening constant, *C* is the strain rate constant, *n* is the strengthening exponent, *m* is the thermal softening coefficient, ${\underline{\varepsilon }}^{p}$ is the effective plastic strain,$\;{\dot{\varepsilon }}^{*}$ is the effective strain rate (dimensionless), ${\dot{\underline{\varepsilon }}}^{p}$ is the strain rate, ${\dot{\varepsilon }}_{0}$ is the reference value for the strain rate, T* is the homologated temperature (dimensionless), *T_room_* is room temperature, *T_melt_* is the melting point, and *T* is the current temperature.

To develop the material models, CurveFitter 2024 was used as a supporting program. Thanks to this, the strength coefficients of the Johnson-Cook material model (Equation (1)) were developed based on the results of the static tensile test. The parameters determined on the basis of experimental data are presented in the table below (Table 2). In the model, since the tests were carried out at a constant room temperature, the component related to the effect of temperature on the mechanical properties of the tested plastics was not considered.

Limit strain with the smoothed particle hydrodynamics (SPH) distortion model was adopted as the failure model. The selection of the smoothed particle hydrodynamics (SPH) method as a tool for modeling damage in materials subjected to significant deformations was dictated by the limitations inherent in classical Lagrangian elements. In the traditional approach, substantial deformations can lead to degeneration of the finite element mesh, rendering further analysis impossible beyond a critical threshold of element deformation. This situation necessitates the removal of elements that would have undergone significant damage, thus limiting the accuracy of modeling destruction processes. In contrast, the SPH method enables the conversion of traditional volumetric finite elements into a set of particles while maintaining equivalent mass and energy. These particles are capable of more accurately modeling the destruction process for materials subjected to large deformations, allowing for the continuation of simulations without the need to remove elements. This facilitates the dynamic modeling of processes such as cracking, tearing, or material fragmentation. The strain value at breaking of the material corresponds to the average strain value at which the sample was torn during the experimental tests. These values are summarized in the table below (Table 3).

### 2.4. Methodology of SEM Observations

Observations of the state of the fracture surface after strength tests performed in accordance with PN-EN ISO 527-3:2019-01 [31] for the static tensile test were carried out with a HITACHI TM-3000 (Tokio, Japan, Hitachi) (scanning microscope equipped with an EDS/EDX detector. Tests were carried out using the secondary electron (SE) detector at an accelerating voltage of 15 kV. Observations were carried out on samples coated (sputtered) with graphite.

### 2.5. Biological Assessment of Samples

Biological testing of the manufactured materials was aimed at predetermining whether they would release toxic substances into the tissues and cause immunological, toxic, or allergic reactions in the living organism. Investigating the biological properties of materials is crucial for assessing their impact on living organisms and the environment. These methods allow a comprehensive analysis of a material’s interaction with cells, tissues, and organisms, which is important in the design of medical products, pharmaceuticals, cosmetics, and food materials. These studies include the accurate determination of a material’s potential toxicity and possible allergic or carcinogenic effects as well as the identification of interaction mechanisms with living organisms. Understanding these aspects is important to ensure the safe use of materials in different areas of life and develop appropriate standards and regulations for their use.

**Figure 7:**
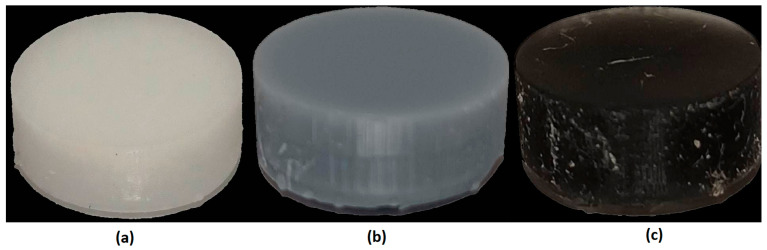
Examples of samples subjected to biocompatibility testing: (**a**) 3D Printing UV-Sensitive Resin; (**b**) ECO UV Resin; and (**c**) 3D Rapid.

A cytotoxicity assessment according to ISO 10993-5:2009 [35] and ISO 10993-12:2021 [36] was used to initially assess the biological properties of the tested materials. Prior to testing, the test samples (six material samples in the form of cylinders with a diameter of 10 mm and a height of 15 mm (Figure 7)) were subjected to steam sterilization (134 °C, 5.5 min). The extract was prepared by immersing the test and control materials (latex and HDPE) in the medium. The L929 cell line was used in the described test system, with tests carried out at 37 ± 1 °C and a CO_2_ concentration of 5 ± 0.1%. The culture medium consisted of MEM supplemented with 10% FBS, 4 mM Glutamax, 100 µ/mL penicillin, 100 µg/mL streptomycin, and 25 µg/mL amphotericin B. Prior to testing, the cells were thawed and multiplied no less than twice. Extraction was performed for 72 ± 2 h at 37 ± 1 °C and an extraction-to-volume ratio of 3 cm^2^/mL. The extracts were unfiltered, centrifuged, or otherwise modified and used up to 24 h after preparation. The extracts were stored in a refrigerator (2–8 °C). Cytotoxicity was then assessed through microscopic observation.

## 3. Results

### 3.1. Static Tensile Test

**Figure 8:**
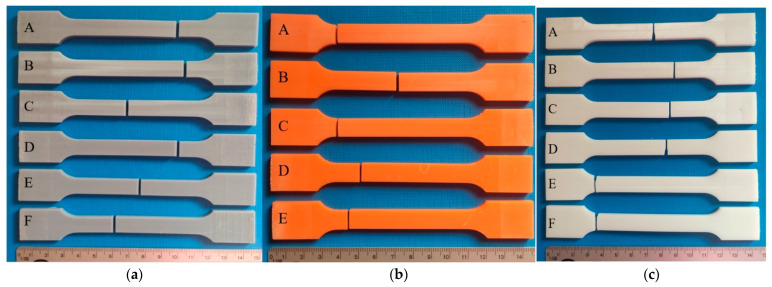
Exemplary sets of samples used to carry out the tensile test: (**a**) ABS-Like; (**b**) Prusament Resin Tough; and (**c**) 3D Printing UV-Sensitive Resin.

**Figure 9:**
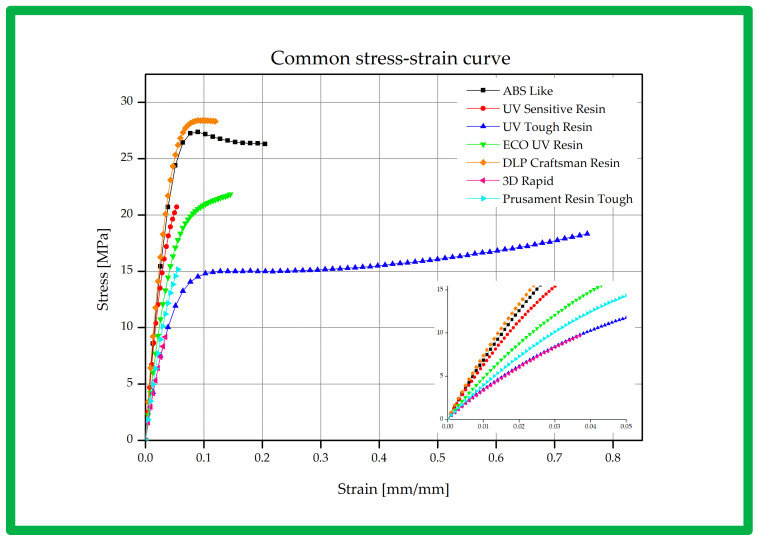
Stress curve for the resins tested.

Depending on the material being tested, different mechanical properties can be determined during a static tensile test. From the diagrams, characteristic points on the stress–strain curve were determined which allowed the assessment of the material’s behavior during loading. These points include the proportional limit, yield strength, and tensile strength. Each of these points reflects specific mechanical characteristics of the material, such as the elasticity, ductility, and strength. The exemplary sets of samples which were tested are shown below (Figure 8), as well as the obtained strength material characteristics (Figure 9) and a summary of the obtained results (Table 4).

In the analysis of the mechanical properties of the tested materials, ABS-Like was found to exhibit elastic-plastic behavior typical of polymeric materials, reaching its highest yield stress of 21.74 MPa, while the 3D Printing UV-Sensitive Resin did not show a clear yield stress, suggesting brittle properties. The UV Tough Resin, ECO UV Resin, and DLP Craftsman Resin also showed the elastic-plastic behavior characteristic of polymeric materials.

In contrast, 3D Rapid and Prusament Resin Tough were characterized by the absence of a clear yield stress, indicating their brittle properties, with 3D Rapid having the lowest yield stress of 3.48 MPa. These observations are significant in the context of material engineering, affecting the potential uses of these materials in various applications, such as the production of components with specific mechanical properties, including in 3D printing and other manufacturing processes.

### 3.2. FEM Analysis

**Figure 10:**
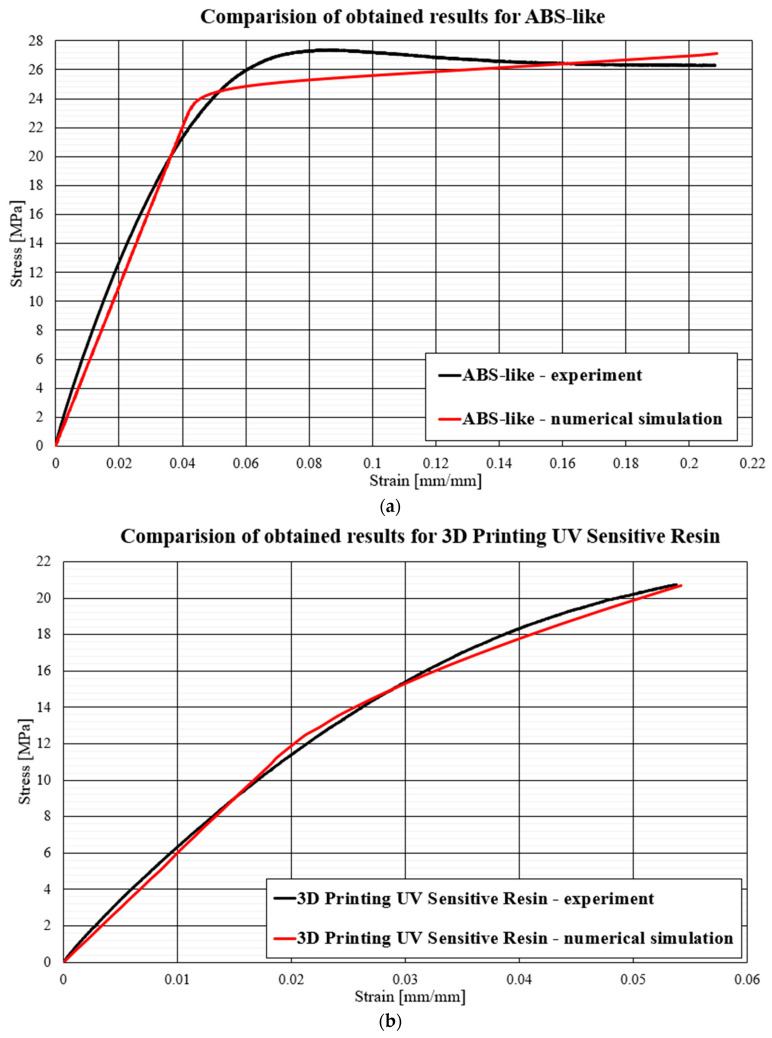

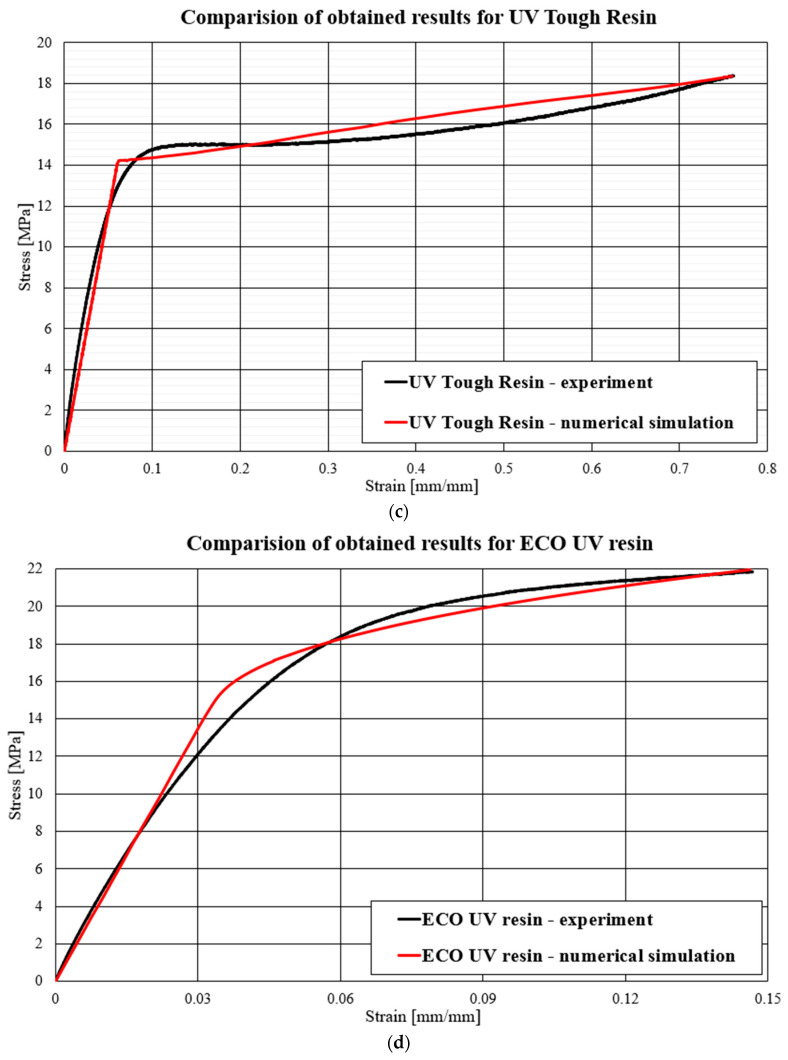

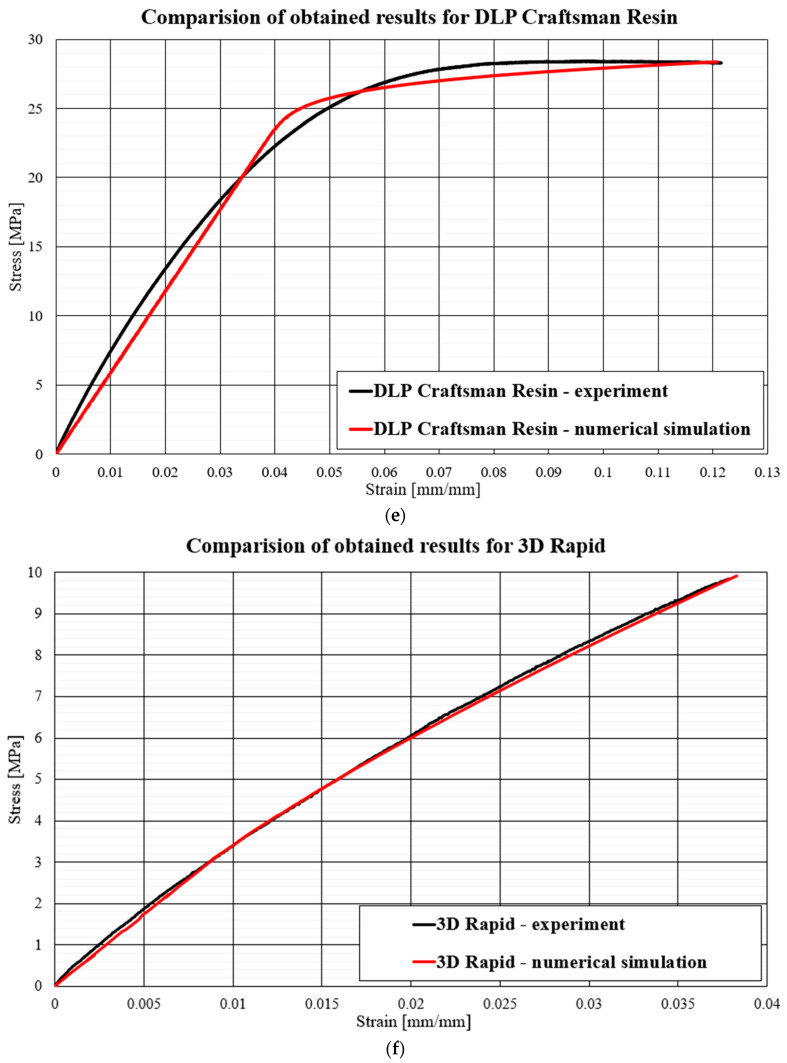

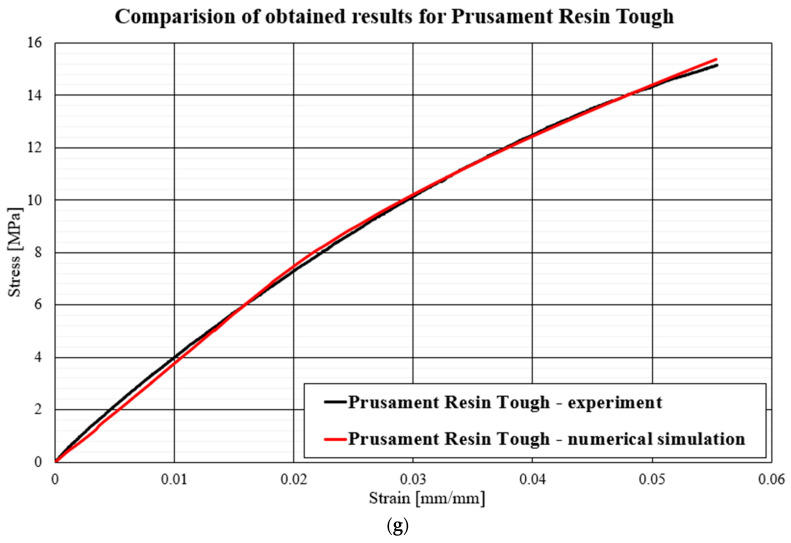
Comparison of obtained results for resins during numerical simulations and experimental results: (**a**) ABS-Like; (**b**) 3D Printing UV-Sensitive Resin; (**c**) UV Tough Resin; (**d**) ECO UV Resin; (**e**) DLP Craftsman Resin; (**f**) 3D Rapid; and (**g**) Prusament Resin Tough.

Below (Figure 10) are graphs comparing the experimental results from the strength tests of the resin samples with the results of the simulation tests in the Abaqus/Explicit computing environment. The results of the tensile test were compared after the application of previously developed parameters in the Johnson-Cook material model.

**Figure 11:**
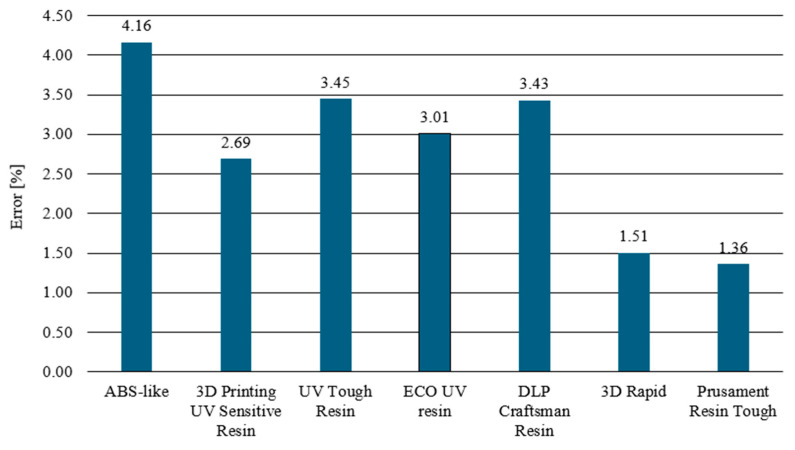
Percentage error obtained from numerical and experimental studies.

To determine the divergence between the experimentally obtained curves and the material characteristics determined by numerical analyses, a method based on identifying the intersection points of the compared curves was used. The intersection points mark the boundaries of the areas for which the area under the curves was calculated by integration. The difference between the values of these areas for each region determines the local divergence between the analyzed curves. The sum of the absolute values of these differences, calculated for all designated areas, provides a measure of the total deviation between the curves. This was then related to the total area under the experimental curve, allowing the resulting discrepancy to be expressed as a percentage, which can be observed below (Figure 11), where all mentioned materials are shown.

The comparative analysis carried out compared the experimental results with their numerical counterparts. Among the tested samples, the best agreement between the experimental curves and numerical models was observed for 3D Rapid, Prusament Resin Tough, and 3D Printing UV-Sensitive Resin materials. The high mapping accuracy, reaching about 98%, was due to the homogeneous plastic strengthening of these materials, as manifested by increasing and monotonic stress curves. Such precise matching was the result of the characteristic power law behavior of the first term of the Johnson-Cook equation. On the other hand, the UV Tough Resin, Eco UV Resin, and DLP Craftsman materials also showed quite good numerical model mapping but with a noticeably higher error rate than the previously mentioned materials. The UV Tough Resin curve was characterized by a lack of monotonicity outside of the elastic range, including the presence of areas of a perfectly plastic character. This resulted in a lack of overlap between the experimental data and the numerical analysis results in the intermediate areas of a complex character. Nevertheless, the model retained significant agreement for key points characteristic of the material, such as the yield and rupture limits. The model, for materials of a similar nature, can be applied in cases where a detailed analysis of the material’s intermediate states is not required, with a focus on the final states, where the fit is sufficient. The Eco UV Resin and DLP Craftsman resin were characterized by an apparent change in behavior once the elastic state was exceeded. This change, however, was subtle and distributed over a non-negligible range of deformation. The adopted material model encountered clear difficulties in fitting well into this region, as is evident in the form of distinct points followed by a sharp change in the slope of the stress–strain curve. The least satisfactory mapping results were obtained for the ABS-Like resin. This was a direct consequence of the nonlinear nature, in the plastic range, of this material. Particularly important here are the areas of plastic softening. Curves in which there are local decreases in stress with the deformation of the specimen cannot be easily determined numerically with the help of the proposed model, the reason for which is the generation of numerical errors.

The Johnson-Cook model shows the highest efficiency in modeling materials characterized by high monotonicity and a proportional increase in stress over the entire strain range. A poorer quality fit is obtained for materials with a pronounced change in behavior beyond the yield point and for materials where the yield point is not clearly delineated. However, it can be used as long as it remains non-decreasing in character and monotonicity for a given elastic and plastic range.

### 3.3. SEM Observations

**Figure 12:**
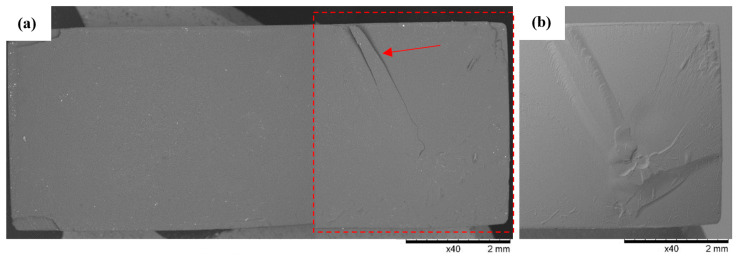
Specimen No. 1A: ABS-Like. SEM: (**a**) global view of the fracture surface obtained after static tensile testing and (**b**) TOPO topographic view of the area marked with a red line.

**Figure 13:**
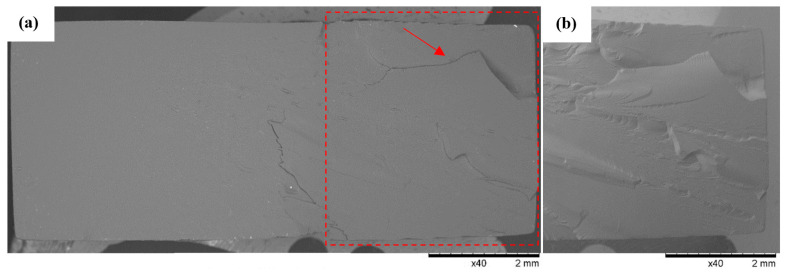
Sample No. 2A: 3D Printing UV-Sensitive Resin. SEM: (**a**) global view of the breakthrough surface obtained after the static tensile sample and (**b**) TOPO topographic mode view of the area marked with a red line.

**Figure 14:**
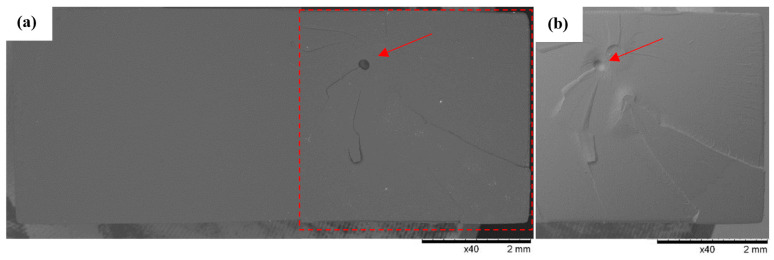
Sample No. 3F: UV Tough Resin. SEM: (**a**) global view of the breakthrough surface obtained after static tensile sampling and (**b**) TOPO topographic mode view of the area marked with a red line.

**Figure 15:**
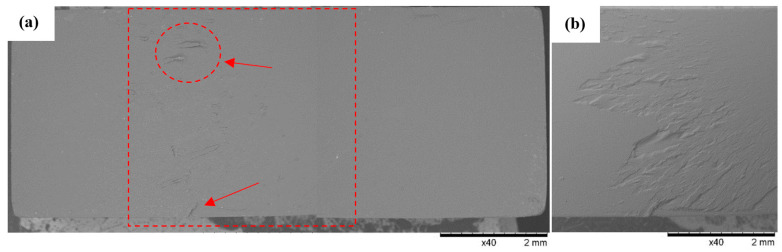
Sample No. 4A: ECO UV Resin. SEM: (**a**) global view of the fracture surface obtained after the static tensile test and (**b**) TOPO topographic mode view of the area marked with a red line.

**Figure 16:**
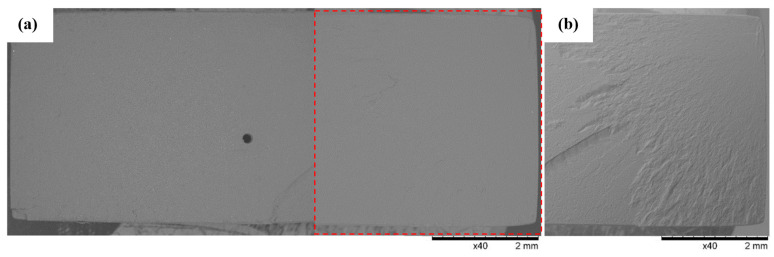
Sample No. 5E: DLP Craftsman Resin. SEM: (**a**) global view of the breakthrough surface obtained after static tensile sampling and (**b**) TOPO topographic mode view of the area marked by the red line.

**Figure 17:**
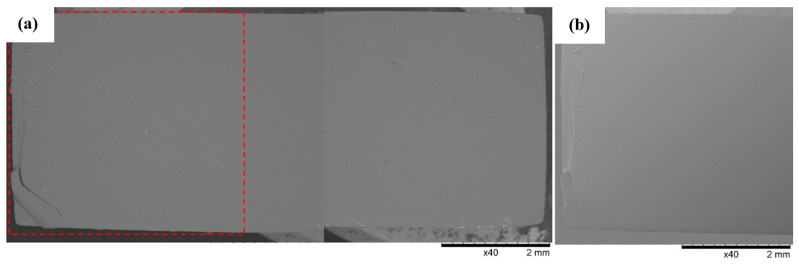
Sample No. 6E: 3D Rapid. SEM: (**a**) global view of the breakthrough surface obtained after static tensile sampling and (**b**) TOPO topographic mode view of the area marked by the red line.

**Figure 18:**
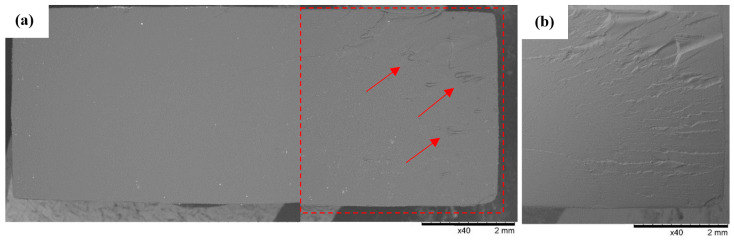
Sample No. 7A: Prusa Orange. SEM: (**a**) global view of the breakthrough surface obtained after static tensile sampling and (**b**) TOPO topographic mode view of the area marked by the red line.

SEM observations of the fracture surfaces were carried out for all the fractures obtained in the static tensile test. Below (Figure 12, Figure 13, Figure 14, Figure 15, Figure 16, Figure 17 and Figure 18), we show representative views of the surface condition of the specimens obtained in each of the seven series of tensile tests on the tested resins.

Example views of the fracture of specimen 1A made from ABS-Like resin is shown below in Figure 12. Observations revealed on the surface of the fracture a system of high faults associated with the passage of the fracture front through an area of local micro-deformation. At the periphery of the specimen, arrangements of smaller faults are clearly visible. Observations of Series 1’s fractures confirmed the phenomenon of detachment of small longitudinal material bands from the fracture surface (indicated by an arrow in the figure).

Localized fracturing of the Series 2 specimens made from 3D Printing UV-Sensitive Resin tended to be more concentrated at one of the shorter edges of the specimen, significantly contributing to the formation of deep craters and faults in this area, as shown in Figure 13. Observations on the surface of the fractures confirmed the phenomenon of detachment of material flakes with sharp edges (indicated by the arrow), indicating the predominance of brittle fractures in the Series 2 specimens.

In the specimens from Series 3’s UV Tough Resin (Figure 14), Series 4’s ECO UV Resin (Figure 15), and Series 5’s DLP Craftsman Resin (Figure 16), the breakthroughs were globally characterized by a flat, regular surface, except for zones in which a defect was occasionally found, the genesis of which was related to the 3D printing process. In the areas where such a defect occurred, effects accompanying local stress concentrations were observed, resulting in the formation of shallow faults on the surface of the breakthrough. For example, Figure 14 shows an area in the vicinity of the air bubble (indicated by the arrow) with radially spreading material faults observed in one of the specimens from Series 3 (3F).

Figure 15 shows a view of the flat faults formed by localized micro-deformations over a small area of the breakthrough surface characteristic of the Series 4 specimens made from ECO UV Resin. Observation of the breakthroughs formed in Series 4 revealed micro-ruptures of the material occurring randomly in all breakthrough zones. In addition, in the areas of micro-ruptures (Figure 15) of the material, small material fragments detaching from the breakthrough surface (indicated by the arrow) were revealed.

A similar pattern of micro-faults was observed at the breakthroughs of the Series 5 samples made from DLP Craftsman Resin (Figure 16) and the Series 7 Prusa Orange samples (Figure 18). The increased concentration of shallow faults tended to be located at the shorter edges of the samples. In addition, the material used in Series 7 (Prusa Orange) showed an increased tendency to fragment (Figure 18), as evidenced by small, detached material elements residing within visible faults (indicated by arrows on the breakthrough view).

SEM observations of the surface of Series 6’s specimens made from 3D Rapid resin revealed the occurrence of a predominantly regular near-flat fracture surface (Figure 17). Possible torn and partially detached material fragments of the fractures in the form of long bands were revealed on the shorter edges of the samples. They mostly formed shallow faults at the edge of the breakthroughs of the samples.

Observation of the fracture surfaces of the specimens after static tension testing made it possible to determine the nature of their destruction and make a general assessment of the state of the fractures. The samples made with 3D printing of resins had a clearly uniform internal structure in which the destruction proceeded with the accentuation of a system of extended faults, indicating a high-energy mode for their fracture (breakthrough formation). It was also found that in the vicinity of printing defects such as blisters, stresses were concentrated locally, thus changing the topography of the breakthrough.

### 3.4. Biological Assessment of Samples

The cytotoxic potential of the tested materials was assessed based on the ISO 10993-5:2009 standard [35]. A numerical grade of three or higher was considered a cytotoxic effect (Table 5). The acceptance criteria were a blank grade <3, positive control grade ≥3, and negative control grade <3.

Based on the results obtained, interpreted in accordance with ISO 10993-5:2009 [36], all tested materials should be considered cytotoxic. The toxicity of the materials tested precludes their use in contact with living organisms. They cannot be used in the manufacture of medical devices, animal contact devices, or in contact with food.

## 4. Discussion and Conclusions

From the mechanical and biocompatibility tests carried out, the basic mechanical parameters of commercially available resins used for domestic use could be determined so that their structures and components could be optimized for specific applications in the future.

Basic parameters such as Young’s modulus and Poisson’s ratio were determined from experimental tests based on uniaxial tensile testing in accordance with ISO 527-3:2019-01 [31]. In addition, because of the use of numerical methods, parameters A, B, and n occurring in the Johnson-Cook material model were also obtained, which in turn were necessary for the numerical analyses. The use of a rheological model based on the above-mentioned equation determined a good match between the experimental and numerical results. By considering the elastic and plastic parts, with the possibility of modifying them by manipulating the strain rate, simple nonlinearities in the materials analyzed could be reproduced under the assumption of a constant temperature.

It was also shown that the use of the SPH method as a model to represent the fracture mechanics of the materials studied, with an assumed boundary strain criterion, showed significant agreement with the experimental results for different types of filaments.

The highest correlation coefficient between the experimental results and those obtained from numerical analyses was obtained for the materials characterized by linearity in the elastic and plastic ranges and by monotonicity in the whole range up to rupturing.

By studying the microstructure of a fracture after the quasi-static tests, it was possible to analyze the type of fracture and its nature. Such an analysis made it possible to determine the fracture’s mechanics and its complexity.

The results of the tests, interpreted in accordance with ISO 10993-5:2009 [35], showed that all samples tested, including ABS-Like, 3D Printing UV, UV Tough, Prusa Orange, 3D Rapid, and Eco UV, were classified as cytotoxic. This classification suggests a potential health risk, which requires further analysis and possible action to minimize the negative effects of these materials on living organisms and the environment.

## Figures and Tables

**Table 1:** Summary of tested SLA-cured resins.

No.	Material	Brand	Density (g/cm^3^)	Tensile Strength (MPa)	Hardness (HS)	**Shrinkage (%)**
1	ABS-Like	AnyCubic	1.15	35–55	84.0	3.72–4.24
2	3D Printing UV-Sensitive	AnyCubic	1.10	36–45	84.5	3.72–4.24
3	UV Tough Resin	AnyCubic	1.15	35–45	84.0	5.10–6.20
4	ECO UV Resin	AnyCubic	1.15	36–52	84.0	1.88–2.45
5	DLP Craftsman Resin	AnyCubic	1.18	35–50	85.0	3.72–4.24
6	3D Rapid	Monocure	1.00	40–50	80.0	0.00–0.50
7	Prusament Resin Tough	Prusa	1.00	30–52	77.5	3.72–4.24

**Table 2:** Obtained resins’ material parameters based on experimental research.

Material	*E* (MPa)	*v* (-)	*ρ* (kg/m^3^)	*A* (MPa)	*B* (MPa)	*n* (-)
ABS-Like	550	0.4	1150	21.74	6.10	0.1586
3D Printing UV Sensitive Resin	600	0.4	1100	11.51	95.46	0.5793
UV Tough Resin	234	0.4	1150	14.23	5.98	6.1872
ECO UV resin	450	0.4	1150	14.89	20.11	0.4304
DLP Craftsman Resin	590	0.4	1180	21.99	11.19	0.2152
3D Rapid	346	0.4	1000	3.01	139.69	0.6478
Prusament Resin Tough	378	0.4	1000	6.98	101.77	0.5903

**Table 3:** Adopted failure strain values for resins.

Material	Failure Strain (mm/mm)
ABS-Like	0.220
3D Printing UV-Sensitive Resin	0.055
UV Tough Resin	0.770
ECO UV resin	0.150
DLP Craftsman Resin	0.123
3D Rapid	0.039
Prusament Resin Tough	0.057

**Table 4:** Summary of results obtained in the tensile test.

Material		R_H_		R_e_		R_m_	
		Stress (MPa)	Strain (mm/mm)	Stress (MPa)	Strain (mm/mm)	Stress (MPa)	Strain (mm/mm)
1.	ABS-Like	21.42 ± 1.14	0.04 ± 0.0029	21.70 ± 1.04	0.04 ± 0.0030	26.31 ± 0.75	0.20 ± 0.052
2.	UV-Sensitive Resin	0.01 ± 0.00	0.01 ± 0.0001	4.52 ± 0.41	0.01 ± 0.0006	20.74 ± 1.71	0.05 ± 0.002
3.	UV Tough Resin	6.06 ± 0.29	0.02 ± 0.0041	6.52 ± 0.28	0.02 ± 0.0041	18.36 ± 1.85	0.76 ± 0.082
4.	ECO UV Resin	7.61 ± 0.69	0.01 ± 0.0008	8.14 ± 0.73	0.02 ± 0.0008	21.84 ± 2.45	0.14 ± 0.055
5.	DLP Craftsman Resin	12.86 ± 0.79	0.02 ± 0.0003	13.38 ± 0.76	0.02 ± 0.0021	28.28 ± 1.66	0.12 ± 0.023
6.	3D Rapid	3.28 ± 0.33	0.01 ± 0.0001	3.48 ± 0.33	0.01 ± 0.0009	9.84 ± 0.95	0.03 ± 0.002
7.	Prusament Resin Tough	6.07 ± 0.28	0.01 ± 0.0005	6.18 ± 0.25	0.02 ± 0.0008	15.17 ± 2.28	0.05 ± 0.015

**Table 5:** Results of cytotoxic potential assessment.

	ABS-Like	3D Printing UV Sensitive	UV Tough Resin	ECO UV Resin	DLP Craftsman Resin	3D Rapid	Prusament Resin Tough
Blank	0	0	0	0	0	0	0
Negative Control	0	0	0	0	0	0	0
Positive Control	4	4	4	4	4	4	4
Test Item	4	4	4	4	4	4	4
	Cytotoxic	Cytotoxic	Cytotoxic	Cytotoxic	Cytotoxic	Cytotoxic	Cytotoxic

## Data Availability

The raw data supporting the conclusions of this article will be made available by the authors on request.
